# Development of a GPU-Accelerated NDT Localization Algorithm for GNSS-Denied Urban Areas

**DOI:** 10.3390/s22051913

**Published:** 2022-03-01

**Authors:** Keon Woo Jang, Woo Jae Jeong, Yeonsik Kang

**Affiliations:** Department of Automotive Engineering, Kookmin University, 77 Jeongneung-ro, Seongbuk-gu, Seoul 02707, Korea; rjsdn8769@kookmin.ac.kr (K.W.J.); wjddnwo95@kookmin.ac.kr (W.J.J.)

**Keywords:** autonomous vehicle, NDT, localization, ROS, 3D LiDAR, GPGPU

## Abstract

There are numerous global navigation satellite system-denied regions in urban areas, where the localization of autonomous driving remains a challenge. To address this problem, a high-resolution light detection and ranging (LiDAR) sensor was recently developed. Various methods have been proposed to improve the accuracy of localization using precise distance measurements derived from LiDAR sensors. This study proposes an algorithm to accelerate the computational speed of LiDAR localization while maintaining the original accuracy of lightweight map-matching algorithms. To this end, first, a point cloud map was transformed into a normal distribution (ND) map. During this process, vector-based normal distribution transform, suitable for graphics processing unit (GPU) parallel processing, was used. In this study, we introduce an algorithm that enabled GPU parallel processing of an existing ND map-matching process. The performance of the proposed algorithm was verified using an open dataset and simulations. To verify the practical performance of the proposed algorithm, the real-time serial and parallel processing performances of the localization were compared using high-performance and embedded computers, respectively. The distance root-mean-square error and computational time of the proposed algorithm were compared. The algorithm increased the computational speed of the embedded computer almost 100-fold while maintaining high localization precision.

## 1. Introduction

Since the mid-1980s, remarkable research efforts and investments have been devoted to developing autonomous driving technology [1,2]. Automotive companies have dedicated remarkable efforts to the commercialization of this technology, which requires near-perfect safety conditions [3,4,5]. Therefore, precise real-time perception of the surrounding environment using various sensors, such as a global navigation satellite system (GNSS), cameras, light detection and ranging (LiDAR) equipment, radio detection and ranging (RADAR) technology, and the inertial measurement unit (IMU), is essential [6]. Diverse types of information are required for safe autonomous driving, including the location of the ego vehicle, road environment, and spatial relationship between the surrounding objects. The techniques used for extracting such information from the environment are interdependent. In addition, as most perception methods are performed under the assumption that the pose of the ego vehicle is accurately known, inaccurate pose information may result in the deterioration of the performance of the autonomous driving system. Although accurate information regarding the position of a vehicle can be obtained using GNSS technology [7,8,9,10], the localization error recommended by ISO 17572 (within 25 cm) cannot be guaranteed in all driving areas [11,12]. In particular, inaccurate GNSS location measurement occurs in GNSS-denied areas, such as urban canyons, overpasses, tunnels, and underpasses, where large objects obscure the sensor receiver [13]. To address this, the use of multiple sensors that can recognize the surrounding environment of a vehicle, rather than the singular use of GNSS technology, is essential for seamless localization [14,15,16].

Cameras have been widely used as sensors in autonomous vehicles [17,18,19,20], and numerous studies have investigated the efficacy of deep learning techniques for processing a large amount of image data. However, as an image captured by a camera records the environment in a two-dimensional (2D) aspect, it cannot be used to accurately estimate the three-dimensional (3D) environment. Cameras are therefore not suitable for establishing precise localization for autonomous driving. Conversely, LiDAR has attracted attention as a sensor for autonomous vehicles because of its ability to acquire 3D distance information from the environment with error rates within a centimeter. Another localization method based on simultaneous localization and mapping [21,22,23] has attracted attention because of its ability to simultaneously estimate the position of a vehicle and create a relevant map. However, the error accumulation that occurs during the estimation process may lead to distortion of the generated map [24]. Although this error accumulation can be corrected using a factor graph-based loop closure [25,26,27], this closure may lead to an abrupt correction in the position of the vehicle, which can result in a dangerous change in the route of an autonomous vehicle in a complex urban environment. In this study, we assumed that a precise map already existed and could be used for the localization of autonomous vehicles via a comparison with current LiDAR measurements [28,29,30].

Recent developments in LiDAR with improved spatial resolution include the 128-channel 3D LiDAR, which can measure more than 400,000 points in a single scan at an update rate of 10–20 Hz. To use such high-resolution LiDAR measurements for localization, a large point cloud must be rapidly processed to ensure precise control of the autonomous vehicle [31]. The normal distribution transform (NDT), a technique that represents a point cloud with a normal distribution, can enable a more compact spatial representation of a point cloud [32]. Therefore, this study used a vector-based normal distribution (ND) map [33]. Although the ND map for localization was represented compactly, the LiDAR still produced numerous measurements, which had to be rapidly and efficiently processed to enable real-time localization. To effectively accelerate the computational speed, this study utilized graphics processing unit (GPU) parallel processing.

The main contributions of this paper can be briefly summarized as follows:

- The proposal of a real-time localization algorithm applying GPU parallel processing to a vector-based normal distribution transform;
- Verification of the algorithm using both the nuScenes dataset (Motional, Boston, Massachusetts, United States) and the CarMaker simulation (IPG Automotive GmbH, Karlsruhe, Baden-Württemberg, Germany) to confirm that the algorithm could operate in a real urban environment and that continuous real-time localization was possible.

The rest of this paper is organized as follows. In Section 2, a method for converting a 3D point cloud map into a vector-based ND map and an efficient map-matching algorithm using GPU parallel processing for localization are described. The performance of the proposed algorithm is verified using an open dataset and simulation. The details of the verification method are described in Section 3. The performance results of the algorithm are discussed in Section 4, and the conclusion of this study is presented in Section 5.

## 2. Methods

**Figure 1:**
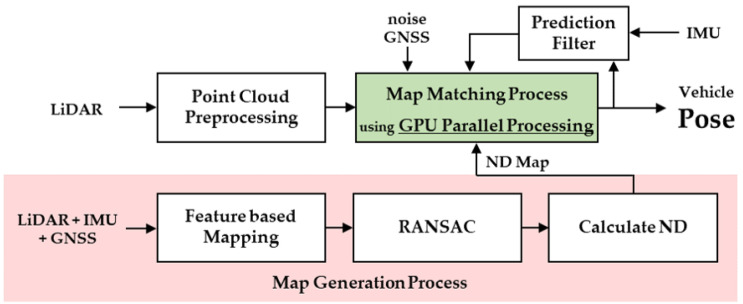
Flowchart of GPU-accelerated NDT localization algorithm.

Figure 1 demonstrates a flowchart of the proposed algorithm. The red area in the figure shows the ND map generation process, which utilized measurements from three sensors (LiDAR, GNSS, and IMU) to create the map, based on universal transverse mercator (UTM) coordinates. The 3D point cloud mapping was performed based on the features of the LiDAR measurement, with the precision increased via calibration using the IMU/GNSS measurements. Subsequently, the point cloud map was converted into an ND map and used for the real-time localization process. Following the point cloud preprocessing, the pose of the ego vehicle was computed by comparing the ND map and the preprocessed point cloud. In addition, the GPU parallel processing was conducted during the map-matching process. Figure 1 demonstrates the GPU-accelerated NDT localization algorithm flow. The resultant pose of the vehicle was entered into a prediction filter to reduce the computational time by predicting the next pose.

### 2.1. Map Generation

#### 2.1.1. Global 3D Point Cloud Map

In this section, we describe the 3D point cloud map generation process. Although the 3D point cloud map generation is not the core focus of this study, it is a preliminary step that is required to create a lightweight map for localization. As the precise 3D point cloud map generation process has been extensively described in various papers, we will only briefly discuss this process.

**Figure 2:**
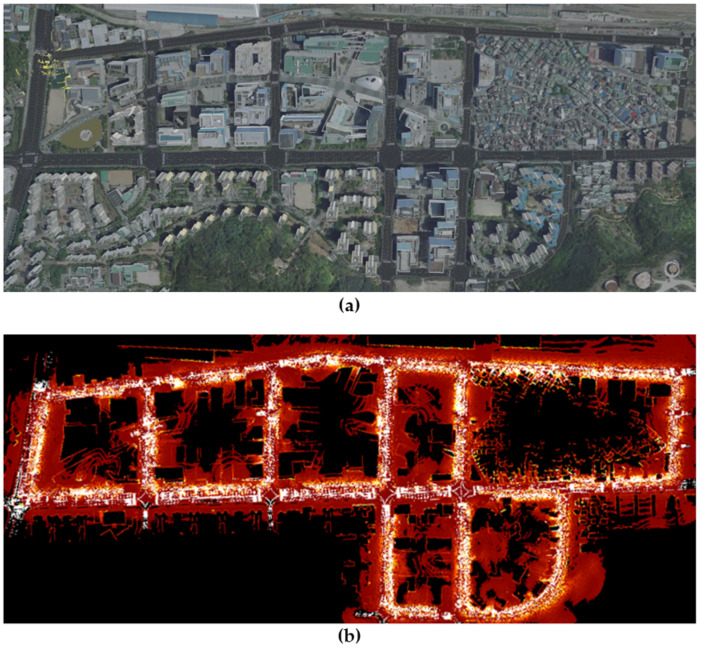
Bird’s-eye views of the urban area implemented in the CarMaker simulation. (**a**) CarMaker simulation viewer. (**b**) 3D point cloud map.

The process was developed on the basis of the LiDAR-based SLAM algorithm, i.e., LIO-SAM, proposed by Tixiao Shan [34]. However, unlike LOAM, scan matching is conducted using LiDAR features comprising planes and edges [35,36] and IMU tracking. The accumulated errors caused by odometry tracking were eliminated by loop closure, and factor graph searching efficiency was improved using GNSS data. In this study, the 3D point cloud map generation algorithm was produced by modifying the LIO-SAM algorithm according to the research environment. Figure 2 demonstrates the 3D point cloud map of the urban area implemented in the simulation.

**Figure 3:**
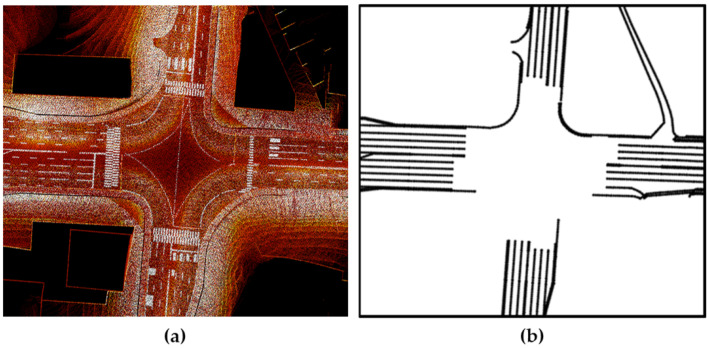
Comparison of road lane information. (**a**) 3D point cloud map. (**b**) High-definition road map.

To verify the 3D point cloud map generation, lane information from a high-definition road map provided by the National Geographic Information Institute, which had a maximum error of 0.25 m, was utilized [37]. The LiDAR sensor returned 3D coordinates (x, y, z) and reflection (intensity) data; lane information was extracted from the intensity measurements. The coordinate system of the point cloud map was converted into UTM coordinates to allow for a comparison between the common lane information in the two maps. Figure 3 demonstrates the comparison of the intensity measurements of the point cloud map and the lane information from the high-definition road map.

#### 2.1.2. Vector-Based Normal Distribution Transform

**Figure 4:**
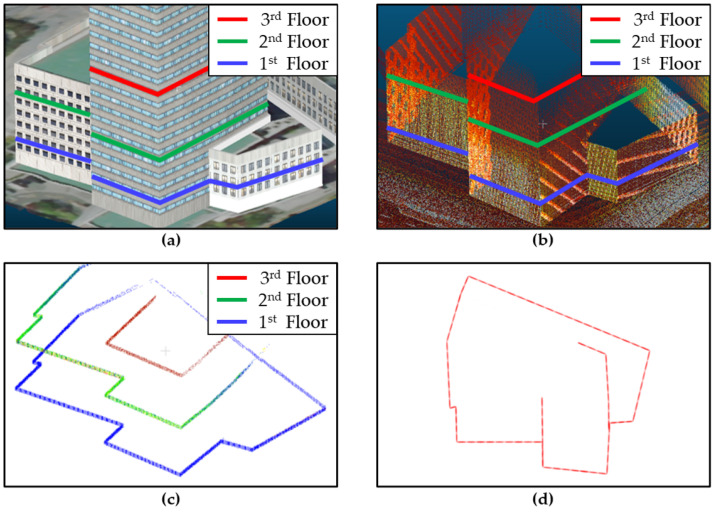
Vector-based NDT process of representing urban buildings. (**a**–**c**) Representative building layers. (**d**) ND map of the building.

The generated 3D point cloud map extensively described the 3D information of the urban area. Generally, an autonomous driving algorithm requires a three-degree-of-freedom vehicle pose comprising x, y, and yaw, which can be obtained using a 2D map. Therefore, in this study, a 2D ND map was created from the 3D point cloud map. The location and shapes of buildings in an urban area are constant, and the location of autonomous vehicles can be uniquely identified by utilizing the shapes of these buildings using LiDAR measurements. However, some buildings in urban areas have complex 3D shapes. Therefore, multiple layers should be included in the map to represent the 3D shape of buildings, with each layer representing the shape of the cross-sections projected in the 2D plane, thereby enabling multiple layers to capture the 3D shape of a building in a 2D map. Figure 4 shows the vector-based NDT of three representative layers of a set of urban buildings.

After creating the 2D point cloud map, the vectors corresponding to the walls of the buildings were extracted using the random sampling consensus (RANSAC) process. During the RANSAC process, the average vertical distance between each vector and the proximate points of the point cloud were represented using an uncertainty parameter (σ), which can be calculated as follows:

(1) $$\sigma =\frac{1}{n}\sum_{k=1}^{n}\;\mathrm{Distance}\;\left(\vec{v},P_{k}\right)$$

where $\vec{v}$ is a vector obtained using the RANSAC process and $P_{k}$ represents summed over positions of the point cloud near the vector. If the vector component was not properly extracted with RANSAC because of noise in the point cloud, the vector was directly designated. The vector and uncertainty information were used to calculate the covariance matrix, which represents the point cloud through NDT, using Equation (2) as follows:

(2) $$\Sigma =U\Lambda U^{T}=\left[\begin{array}{cc} \lambda_{1}v_{x}^{2}+\lambda_{2}n_{x}^{2} & \lambda_{1}v_{x}v_{y}+\lambda_{2}n_{x}n_{y}\\ \lambda_{1}v_{x}v_{y}+\lambda_{2}n_{x}n_{y} & \lambda_{1}v_{y}^{2}+\lambda_{2}n_{y}^{2} \end{array}\right]$$

where *U* is a matrix consisting of eigenvectors defined by $\vec{v}$ and its perpendicular vector, $\vec{n}$, as follows:

(3) $$U=\left[\begin{array}{cc} \vec{v} & \vec{n} \end{array}\right]=\left[\begin{array}{cc} v_{x} & n_{x}\\ v_{y} & n_{y} \end{array}\right]$$

where Λ is a matrix comprising two eigenvalues, i.e., $\lambda_{1}$ and $\lambda_{2}$. Eigenvalue $\lambda_{1}$ was set as half the length of $\vec{v}$, and $\lambda_{2}$ was set as σ.

(4) $$\Lambda =\left[\begin{array}{cc} \lambda_{1} & 0\\ 0 & \lambda_{2} \end{array}\right]=\left[\begin{array}{cc} L/2 & 0\\ 0 & \sigma \end{array}\right]$$

**Figure 5:**
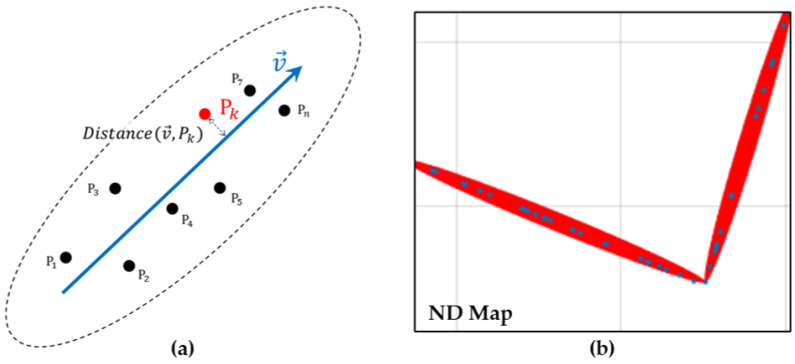
(**a**) Elements used for the generation of the vector-based ND map. (**b**) Part of vector-based ND map and point cloud map.

Figure 5 demonstrates elements of the ND map used in Equations (1)–(4). The blue arrow in Figure 5a represents the vector that was extracted using RANSAC, and the black points on both sides of the arrow represent the point cloud near the vector. Figure 5b shows an approximation of the point cloud, as measured from the building wall by the ND map. The covariance matrix, i.e., Σ, and the center point of $\vec{v}$, $V_{c}$ were stored together in the vector-based ND map file. The 3D point cloud map with an original size of 2 GB was reduced to an ND map with a size of 2 MB using the vector-based NDT process.

### 2.2. Map Matching Using GPU Parallel Processing

This study developed a real-time localization algorithm by comparing an ND map with LiDAR measurements. To do so, the LiDAR measurements required several preprocessing steps. First, the points in the cloud up to a height of 4 m from the road’s surface were removed to eliminate unnecessary measurements. This preprocessing method rapidly removed measurements originating from the ground and dynamic objects. Subsequently, a 3D point cloud containing only the building information was projected onto a 2D x–y plane. In addition, the real-time LiDAR measurements were not downsampled, thus enhancing the accuracy of the localization result.

The preprocessed 2D point cloud was transferred to the UTM coordinate system using the vehicle pose (position and heading angle). The 2D homogeneous matrix that was used for the transformation of the coordinate is shown in Equation (5).

(5) $$Pose=\left[\begin{array}{c} x_{t}\\ y_{t}\\ \theta_{r} \end{array}\right],\;M_{t}=\left[\begin{array}{ccc} \cos\theta_{r} & -\sin\theta_{r} & x_{t}\\ \sin\theta_{r} & \cos\theta_{r} & y_{t}\\ 0 & 0 & 1 \end{array}\right]$$

The transformed point cloud, i.e., $M_{t}P_{k}$, was used to determine the nearest vector, $v_{j}$, in the ND map. Subsequently, the score was calculated using Equation (6), where $C_{j}$ represents the center point of $v_{j}$ and $\Sigma_{j}$ represents the covariance matrix of $v_{j}$.

(6) $$\begin{array}{cc} \mathrm{Score}\left(M_{t},P_{k},j\right) & =\exp\left(-\frac{{\left(M_{t}P_{k}-C_{j}\right)}^{T}\Sigma_{j}^{-1}\left(M_{t}P_{k}-C_{j}\right)}{2}\right) \end{array}$$

After the score was calculated, the Cost for each $M_{t}$ was calculated by summing over the scores, as shown in Equation (7). The negative sign was applied to modify the sum of scores as a minimization problem.

(7) $$\mathrm{Cost}\left(M_{t}\right)=-\sum_{k=1}^{n}\;\mathrm{Score}\;\left(M_{t},P_{k},j\right)$$

Thereafter, Newton’s method for optimization was applied to the Cost to repeatedly correct the pose toward the minimum value of the Cost. The calculation of the change in the pose, i.e., ΔPose, used a Hessian/gradient matrix, as expressed in Equation (8) below.

(8) $$\Delta Pose=\left[\begin{array}{c} \Delta x_{t}\\ \Delta y_{t}\\ \Delta \theta_{r} \end{array}\right]=-H^{-1}g,\;H=\left[\begin{array}{ccc} \frac{\partial^{2}C}{\partial x^{2}} & \frac{\partial^{2}C}{\partial x\partial y} & \frac{\partial^{2}C}{\partial x\partial \theta }\\ \frac{\partial^{2}C}{\partial y\partial x} & \frac{\partial^{2}C}{\partial y^{2}} & \frac{\partial^{2}C}{\partial y\partial \theta }\\ \frac{\partial^{2}C}{\partial \theta \partial x} & \frac{\partial^{2}C}{\partial \theta \partial y} & \frac{\partial^{2}C}{\partial \theta^{2}} \end{array}\right],\;g=\left[\begin{array}{c} \frac{\partial C}{\partial x}\\ \frac{\partial C}{\partial y}\\ \frac{\partial C}{\partial \theta } \end{array}\right]$$

**Figure 6:**
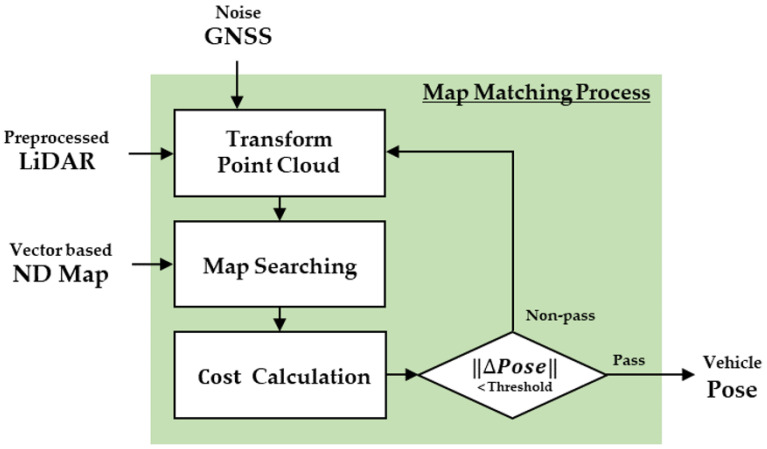
Flowchart of the map-matching process.

Figure 6 demonstrates a flowchart of the map-matching process between the ND map and the point cloud. The map-matching process was performed for all points in the cloud and was repeated until an appropriate pose was obtained. The computation time of the overall map-matching process increased with an increase in the number of point clouds. To reduce the overall computation time, this study used GPU parallel processing for the map-matching process.

For GPU parallel processing, a large amount of the point cloud was moved from the central processing unit (CPU; Host) memory to the GPU (Device) memory, after which the operations on each point were performed parallelly in the GPU thread. This ensured that an operation that needed to be repeated N times on the CPU could be executed at once on the N threads of the GPU. As the size of data to be computed increased, the efficiency of the GPU parallel processing increased compared with that of the CPU serial processing [38]. However, not all operations were suitable for parallel processing. Therefore, to take advantage of parallel processing, the algorithm should comprise numerous simple iterative operations rather than a small number of complex branching operations. The localization algorithm proposed in this study was suitable for GPU parallel processing as the same operations, Equations (5)–(8), were repeated for all points $P_{k}$ in the point cloud. However, when a large number of individually calculated values from all point clouds had to be added to obtain a single value, bottlenecks were observed in the map-matching process.

#### 2.2.1. GPU Parallel Reduction

**Figure 7:**
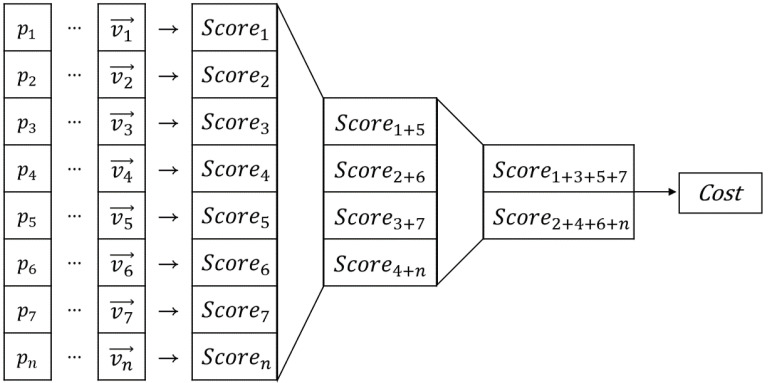
Operational flow of the parallel cost calculation.

In this study, the bottlenecks of the map-matching process were resolved using parallel reduction [39]. Figure 7 shows the computational flow of a cost calculation in which the parallel reduction was applied. To utilize CPU serial processing for the cost calculation, the addition of the score value to the cost value was repeated consecutively. In contrast, the parallel reduction performed a tree-type addition to obtain the final overall sum by repeating the individual additions of two pieces of data (Figure 7). The parallel reduction process reduced the number of operations conducted from N to $\log_{2}N$. Algorithm 1 represents the pseudocode of the parallel cost calculation. Different GPU threads were used to simultaneously compute the score between each point and the nearest vector. Next, threads that were separated by a specific index were added together. These computations were conducted for only half of the total threads, and the number of threads subjected to these computations was continuously reduced by half to ensure that the operation was conducted only on the first thread. Following the parallel reduction, the sum of all the scores was computed.

When the parallel reduction was performed, the score data were repeatedly loaded. However, storage of the score data in the global memory resulted in unnecessary repetition of the data transfer delay between the thread and the global memory during operation [40]. Therefore, the score data were transferred from the global memory to the shared memory in advance to minimize the delay caused by data loading.

**Algorithm 1:** Parallel Cost Calculation

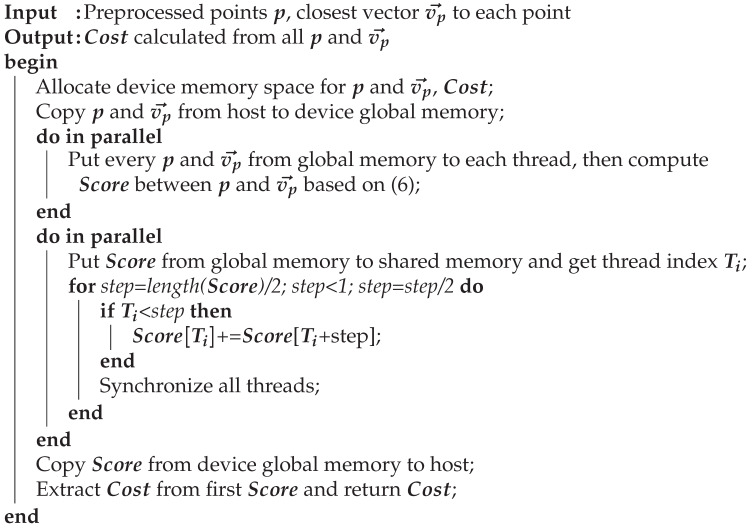



**Figure 8:**
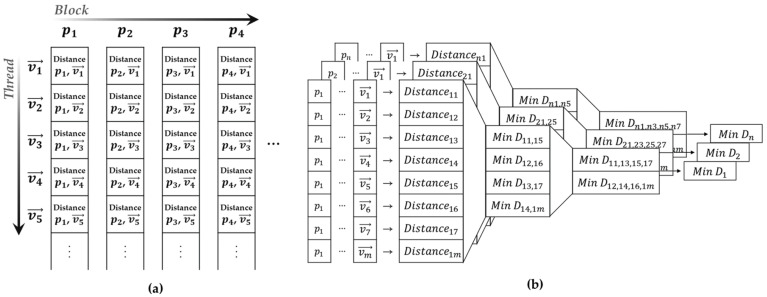
(**a**) Depiction of using GPU thread and block. (**b**) Operational flow of the parallel map searching.

Before conducting the cost calculation, map searching was conducted to determine the closest vector to each point. A parallel reduction was applied to the comparison operation during this process. In contrast to the cost calculation, a parallel reduction was conducted separately for each point. As shown in Figure 8a, different points are loaded in each GPU block, and the distances from these points to all vectors are calculated. Subsequently, parallel reduction was individually conducted for each block, as shown in Figure 8b. Thereafter, the nearest vector for each block was calculated using the map-searching results. Algorithm 2 represents the pseudocode of the parallel map searching.

**Algorithm 2:** Parallel Map Searching

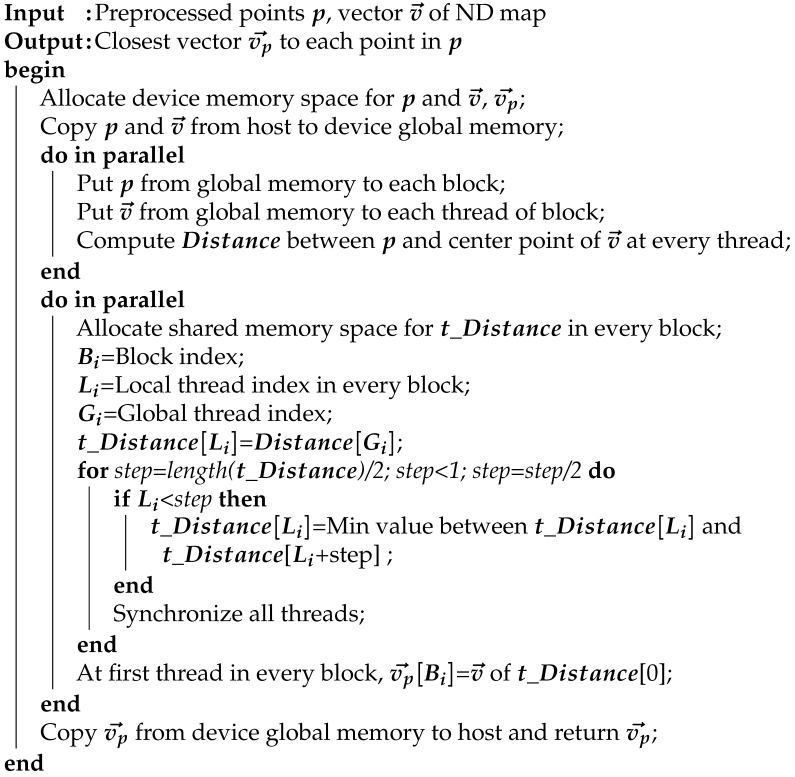



## 3. Experiments

In this study, the proposed algorithm was developed on the basis of a C/C++ robot operating system in Ubuntu 18.04 (Canonical Ltd., London, UK). The computation was accelerated using the GPU parallel processing abilities of the CUDA platform (NVIDIA Corporation, Santa Clara, CA, USA). Table 1 shows the detailed specifications of the computing system used for verification.

The proposed vector-based normal distribution, i.e., VNDT, was compared with other localization methods—specifically, iterative closest point, i.e., ICP, and grid-based normal distribution transform, i.e., GNDT, [32,41]. In this study, we used corresponding algorithms that were implemented on the Point Cloud Library [42]. The tuning parameters common to all algorithms were the maximum number of iterations and the iteration tolerance. The maximum number of iterations was set to 50, and the iteration tolerances were set to 0.1 cm and 0.01${}^{\circ }$. Detailed descriptions of the tuning parameters for these algorithms are available in [43,44]. In addition, the grid size and vector length are important tuning parameters for NDT algorithms. In this study, these parameters were set to 2 m.

### 3.1. Open Datasets

To verify the performance of the algorithm with real sensor data, including noise, the nuScenes dataset was used [45]. The nuScenes dataset is a public dataset for autonomous driving developed by an autonomous vehicle company, Motional. Scenes containing complex driving environments in Boston and Singapore were selected from the dataset. The reasons for choosing each scene are as follows.

- scene-0061: The scene data were recorded from Singapore’s One North. The driving route at an intersection was under construction. Using the scene data, the accuracy of localization in a congested situation (e.g., passersby standing before traffic signals, construction machine on the road) was verified.
- scene-0103: The scene data were recorded from Boston Seaport. The driving route started at the Congress Street Bridge with no buildings nearby. Using the scene data, the initialization of localization was verified with distant building information.
- scene-1094: The scene data were recorded from Singapore’s Queenstown. Since the vehicle was driven on a rainy road at night, the LiDAR measurement was unstable. Using the scene data, the accuracy of localization was verified using the unstable LiDAR measurement.

**Figure 9:**
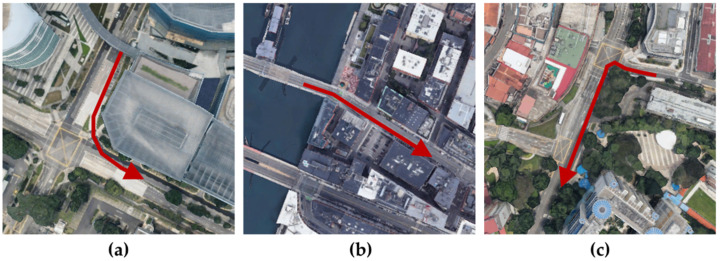
Driving routes (i.e., red arrow) of the selected nuScenes scenes. (**a**) scene-0061, (**b**) scene-0103, and (**c**) scene-1094.

Each scene included ego pose data that had been precisely calibrated using a point cloud map provided by Motional. In this study, the ND map was produced by stacking point cloud data according to the corresponding ego pose. The driving time for acquiring each scene’s data was 20 s, and the data from each sensor (LiDAR, GPS, IMU, etc.) and the calibrated ego pose were stored at a constant rate of 2 Hz. The driving distances for each scene were 91 m (scene-0061), 118 m (scene-0103), and 126 m (scene-1094). Figure 9 demonstrates the driving route of each scene.

### 3.2. Simulation

**Figure 10:**
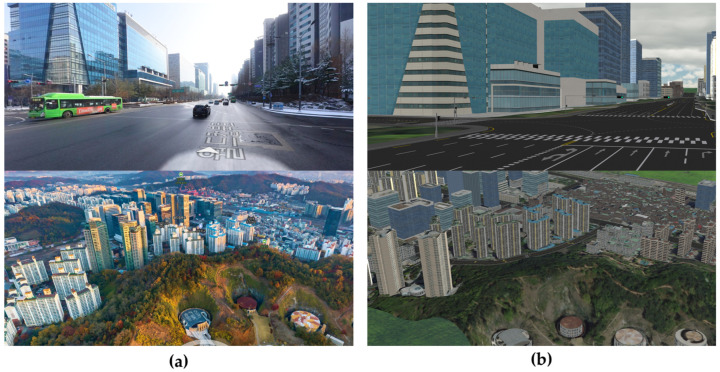
Comparison of the urban area implementation in CarMaker. (**a**) Real urban view. (**b**) CarMaker simulation viewer.

The CarMaker simulation program (IPG Automotive GmbH) was used to verify the computational efficiency with long-term driving. To conduct the localization at the same coordinates as the real urban area, the CarMaker simulation was developed according to the high-definition road map information provided by the National Geographic Information Institute of Korea. It was also possible to implement the simulated buildings with a shape and density very similar to reality by referring to photos of urban areas. Figure 10 shows a comparison of the real and simulated urban areas.

Simulated driving was conducted using a simulation vehicle equipped with LiDAR, GNSS, and IMU sensors in CarMaker. To enhance the realism of the simulation, the vehicle was developed according to the specifications of the KIA Niro. Table 2 lists the detailed specifications.

Table 3 lists the detailed specifications of the simulation LiDAR sensor, which was implemented by referring to Ouster’s OS1-64 model.

**Figure 11:**
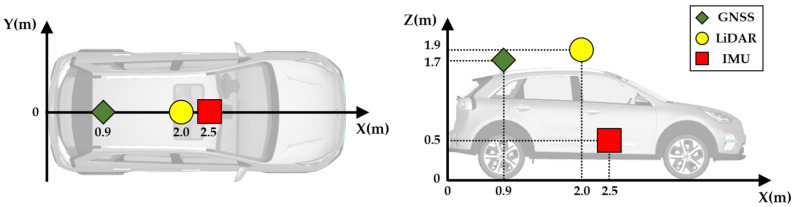
Position of the sensors attached to the simulation vehicle.

Considering that there was no error in the GNSS within the simulation, a radial uniform distribution error of 2 m was introduced. The noise in the GNSS reading was used as the initial pose in the localization algorithm, and the original GNSS was used only to represent the ground truth for evaluating the performance of the algorithm. Figure 11 shows the position of the sensors attached to the simulation vehicle. The LiDAR sensor was placed at the center of the front seat of the vehicle and was installed 15 cm above the vehicle roof to reduce the shadow area. The GNSS sensor was attached to the center of the roof above the rear wheel axle, and the IMU sensor, which was used to track the vehicle odometry, was placed at the vehicle’s center of gravity.

**Figure 12:**
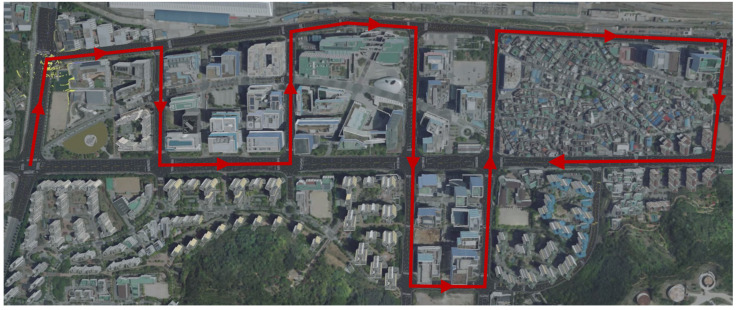
Verification route (i.e., red arrow) of CarMaker.

The length of the verification route was 4300 m, and the total driving time was 7 min. During the simulation, the vehicle moved at a speed of 60 km/h using the CarMaker auto-driving system. Since localization was conducted on all LiDAR inputs (10 Hz), the root-mean-square error (RMSE) was calculated with more than 4000 resultant data points. Figure 12 shows the verification route in CarMaker.

## 4. Results and Discussion

### 4.1. Open Datasets

**Figure 13:**
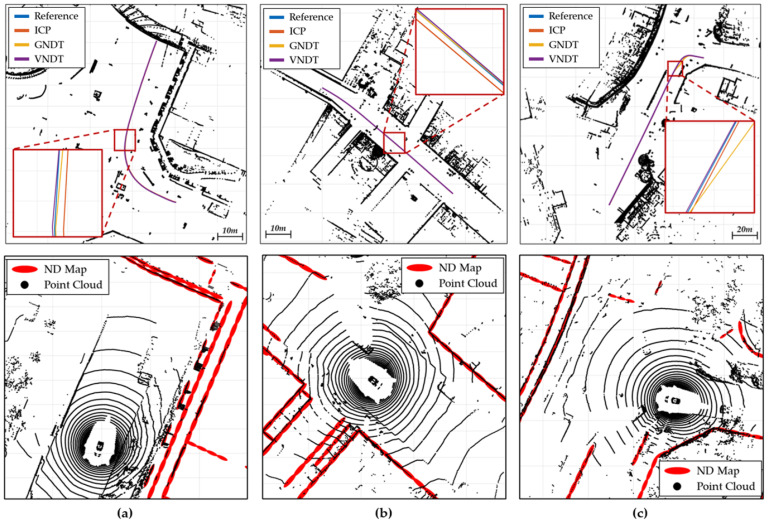
Comparison between the localization results and the reference (**top**) and LiDAR map-matching results (**bottom**) in nuScenes. (**a**) scene-0061, (**b**) scene-0103, and (**c**) scene-1094.

The top row of Figure 13 shows the localization results from the nuScenes dataset. Compared with the reference ego pose, VNDT demonstrated the highest precision among the localization algorithms. In contrast, ICP had a constant bias, and GNDT was adversely affected by noise. The bottom row of Figure 13 shows the matching results of the LiDAR measurement and ND map in nuScenes. The red ellipses in the figure represent the ND information, and the black points represent the LiDAR measurements. The bird’s-eye perspective indicates that the point cloud had been properly matched to each ellipse. Notably, the point cloud was properly matched to the ND map even when the shapes of the buildings were complex and dense.

The RMSE and the maximum localization error were calculated (Table 4). The RMSE in both the longitudinal and lateral directions was within 2 cm, and the heading was within 0.1${}^{\circ }$. The longitudinal and lateral errors were within 25 cm; this meant that the performance of the algorithms did not exceed the localization error value recommended by ISO 17572.

Table 5 lists the average computation time for each localization algorithm. The proposed algorithm (VNDT using GPU) achieved a real-time performance suitable for use with 10–20 Hz LiDAR and increased the computational speed of the embedded computer almost 100-fold. The computation time of the GPU parallel processing functions proposed in Section 2.2.1 was analyzed in a CarMaker simulation for verification because the nuScenes dataset included only 30 data points in one scene.

### 4.2. Simulation

**Figure 14:**
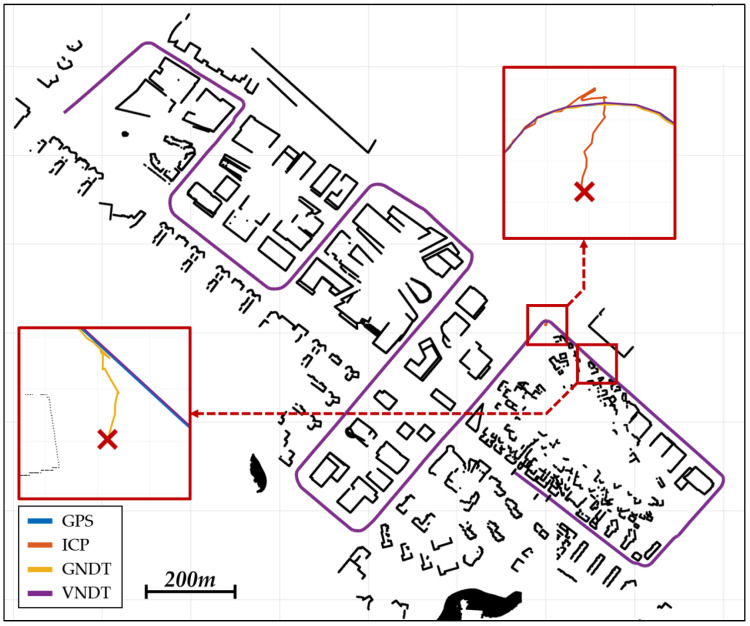
Localization result from the CarMaker simulation.

Figure 14 shows the localization result from the CarMaker simulation. The ICP and GNDT algorithms failed to localize the vehicle in an area where small buildings were densely located. Therefore, it is not possible to analyze the precision and calculation time of the ICP and GNDT algorithms. Table 6 lists the accuracy of the VNDT algorithm.

**Figure 15:**
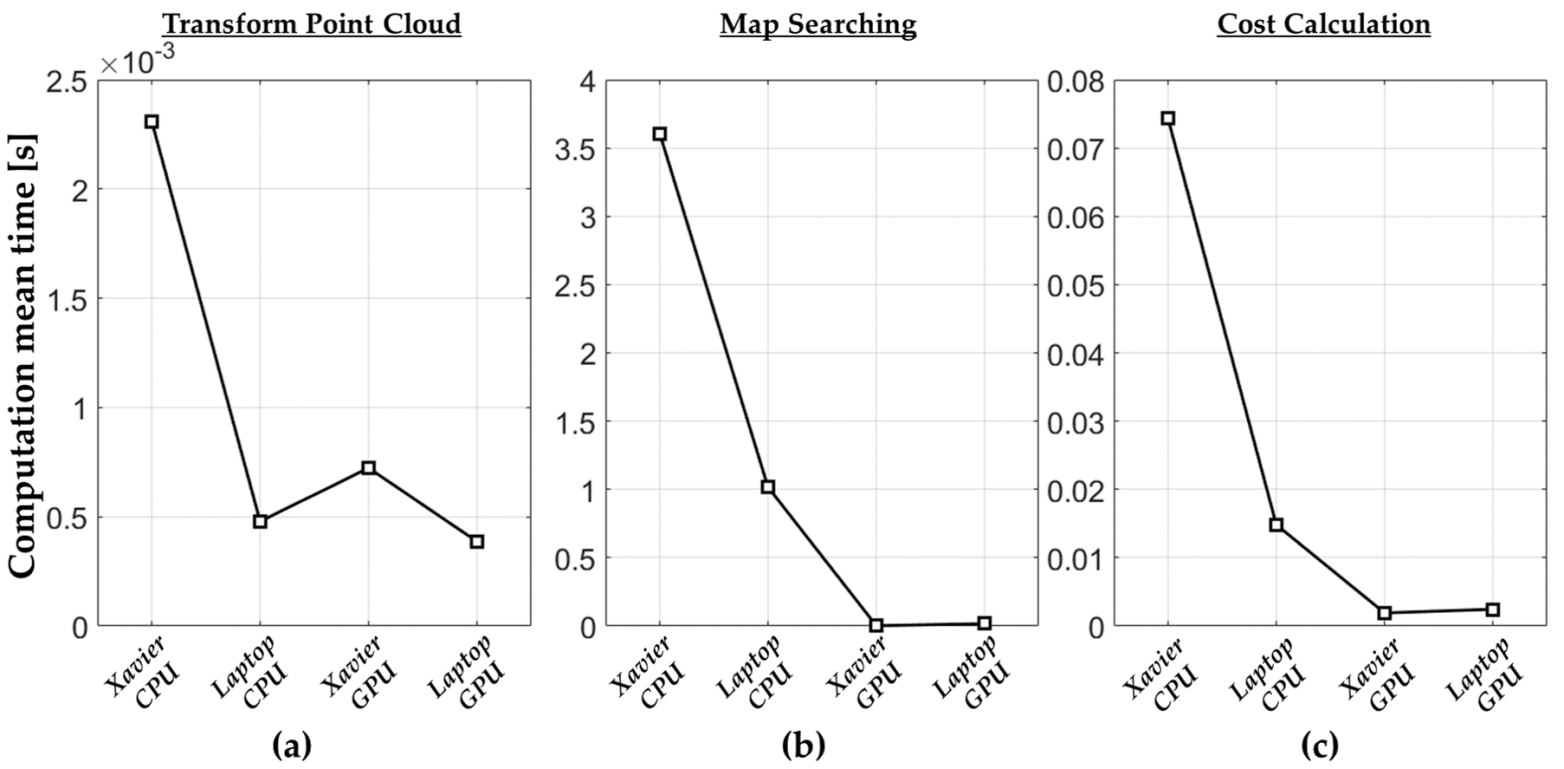
The average computation time for each function in the CarMaker simulation. (**a**) Transform point cloud. (**b**) Map searching. (**c**) Cost calculation.

Table 7 compares the computation time between the CPU and GPU-based VNDT algorithms. Figure 15 shows a comparison of the average computation time of the GPU parallel processing functions (i.e., transform point cloud, map searching, and cost calculation). The computation time of each function includes the length of the data transfer delay between the CPU and GPU memory.

**Figure 16:**
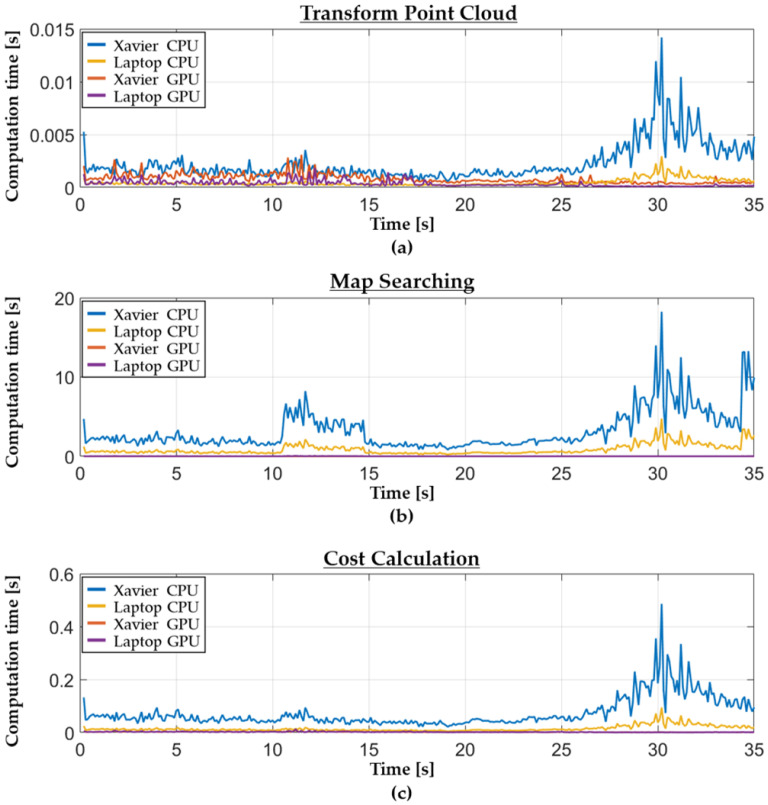
Computation time for each function in the CarMaker simulation. (**a**) Transform point cloud. (**b**) Map searching. (**c**) Cost calculation.

Figure 16 shows the computation time for each function in the driving time. The figure indicates that the computation time using CPU serial processing increased with an increase in the size of the point cloud after preprocessing. In contrast, the amount of data had no remarkable effect on the computational speed of the GPU parallel processing.

## 5. Conclusions

In this study, we introduced a GPU-accelerated vector-based NDT localization algorithm that could guarantee precision and real-time performance for 10–20 Hz high-resolution LiDAR, even in GNSS-denied urban areas. Therefore, a vector-based ND map suitable for the parallel processing algorithm was used for the reference map. Parallel processing was applied to the map-matching process to maintain a stable and fast computational speed, regardless of the size of the LiDAR measurement data. In particular, the parallel reduction was used for map searching and the cost calculation function of the map-matching process. The performance of the proposed algorithm was evaluated with the nuScenes open dataset and urban environment in a CarMaker simulation. The performance of the proposed algorithm satisfied the accuracy required by ISO 17572. In addition, the fast computational speed of the algorithm enabled a localization update rate of 10 Hz. Therefore, the proposed algorithm exhibited enhanced computational speed and highly reliable performance. In a future study, we plan to study localization in the actual urban area that was referenced in the simulation. We will propose a robust localization algorithm for the real environment and analyze the GPU efficiency in detail.

## Figures and Tables

**Table 1:** Specifications of the computing system used for conducting the verification.

	Laptop	NVIDIA Jetson Xavier AGX
CPU	Intel Core i7-9750H	8-core ARM v8.2 64-bit CPU
RAM	16 GB	32 GB
GPU	NVIDIA GeForce RTX2070	512-core Volta GPU
OS	Ubuntu 18.04 + ROS Melodic	
CUDA	Ver.11.2	

**Table 2:** Specified parameters of the simulation vehicle.

Designation	Parameter	Designation	Parameter
Overall Length	4735 mm	Wheelbase	2700 mm
Overall Width	1805 mm	Front Tread	1562 mm
Overall Height	1570 mm	Rear Tread	1572 mm
		Curb Weight	1755 kg

**Table 3:** Specified parameters of the simulation LiDAR.

Designation	Parameter	Designation	Parameter
Vertical Resolution	64 channels	Scan Range	100 m
Horizontal Resolution	1024	Rotation Rate	10 Hz
Vertical Angular Resolution	0.35${}^{\circ }$	Points per Second	655,360
Vertical Field of View	−22.5${}^{\circ }$∼22.5${}^{\circ }$	Data Field	X, Y, Z, Intensity

**Table 4:** Accuracy comparison of the localization methods in nuScenes.

		Localization Error								
		Longitudinal			Lateral			Heading		
		ICP	GNDT	VNDT	ICP	GNDT	VNDT	ICP	GNDT	VNDT
**scene-0061**	**RMSE**	5.57 cm	2.78 cm	**1.40 cm**	7.17 cm	3.16 cm	**1.81 cm**	0.03${}^{\circ }$	0.08${}^{\circ }$	**0.02${}^{\circ }$**
	**MAX. dev.**	12.26 cm	9.16 cm	**4.13 cm**	14.92 cm	11.92 cm	**4.31 cm**	0.09${}^{\circ }$	0.31${}^{\circ }$	**0.06${}^{\circ }$**
**scene-0103**	**RMSE**	3.76 cm	3.27 cm	**1.92 cm**	9.01 cm	2.58 cm	**1.54 cm**	0.02${}^{\circ }$	0.03${}^{\circ }$	**0.02${}^{\circ }$**
	**MAX. dev.**	6.95 cm	12.92 cm	**4.9 cm**	5.04 cm	10.75 cm	**3.39 cm**	0.10${}^{\circ }$	0.13${}^{\circ }$	**0.05${}^{\circ }$**
**scene-1094**	**RMSE**	6.05 cm	20.8 cm	**1.59 cm**	4.76 cm	17.23 cm	**1.44 cm**	0.05${}^{\circ }$	0.10${}^{\circ }$	**0.02${}^{\circ }$**
	**MAX. dev.**	17.44 cm	140.93 cm	**4.58 cm**	11.31 cm	136.31 cm	**6.76 cm**	0.12${}^{\circ }$	0.82${}^{\circ }$	**0.08${}^{\circ }$**

**Table 5:** Computation time comparison of localization methods in nuScenes.

		ICP	GNDT	VNDT(CPU)	VNDT(GPU)
**scene-0061**	**Xavier**	401.98 ms	457.10 ms	4934.22 ms	**51.82 ms**
	**Laptop**	134.76 ms	145.79 ms	1263.21 ms	**28.35 ms**
**scene-0103**	**Xavier**	1036.08 ms	946.25 ms	5723.73 ms	**60.12 ms**
	**Laptop**	340.51 ms	305.23 ms	1387.25 ms	**34.91 ms**
**scene-1094**	**Xavier**	726.92 ms	612.30 ms	4573.10 ms	**47.63 ms**
	**Laptop**	219.88 ms	191.57 ms	1300.39 ms	**28.95 ms**

**Table 6:** Accuracy of the proposed localization method in CarMaker.

	Localization Error		
	Longitudinal	Lateral	Heading
**RMSE**	2.21 cm	2.92 cm	0.12${}^{\circ }$
**MAX. dev.**	6.84 cm	7.38 cm	0.31${}^{\circ }$

**Table 7:** Computation time comparison of localization methods in CarMaker.

	VNDT(CPU)	VNDT(GPU)
**Xavier**	4125.16 ms	**43.81 ms**
**Laptop**	1271.98 ms	**26.11 ms**

## References

[1] Badue C., Guidolini R., Carneiro R.V., Azevedo P., Cardoso V.B., Forechi A., Jesus L., Berriel R., Paixao T.M., Mutz F. (2021). Self-driving cars: A survey. Expert Syst. Appl..

[2] Bimbraw K. Autonomous cars: Past, present and future a review of the developments in the last century, the present scenario and the expected future of autonomous vehicle technology. Proceedings of the 2015 12th International Conference on Informatics in Control, Automation and Robotics (ICINCO).

[3] Schoettle B., Sivak M. (2014). A Survey of Public Opinion about Autonomous and Self-Driving Vehicles in the US, the UK, and Australia.

[4] Lee C., Ward C., Raue M., D’Ambrosio L., Coughlin J.F. (2017). Age differences in acceptance of self-driving cars: A survey of perceptions and attitudes. International Conference on Human Aspects of IT for the Aged Population.

[5] Liu P., Zhang Y., He Z. (2019). The effect of population age on the acceptable safety of self-driving vehicles. Reliab. Eng. Syst. Saf..

[6] Kocić J., Jovičić N., Drndarević V. Sensors and sensor fusion in autonomous vehicles. Proceedings of the 2018 26th Telecommunications Forum (TELFOR).

[7] Hofmann-Wellenhof B., Lichtenegger H., Wasle E. (2007). GNSS–Global Navigation Satellite Systems: GPS, GLONASS, Galileo, and More.

[8] Blomenhofer H., Ehret W., Leonard A., Blomenhofer E. GNSS/Galileo global and regional integrity performance analysis. Proceedings of the 17th International Technical Meeting of the Satellite Division of The Institute of Navigation (ION GNSS 2004).

[9] Kaplan E.D., Hegarty C. (2017). Understanding GPS/GNSS: Principles and Applications.

[10] Yang Y., Mao Y., Sun B. (2020). Basic performance and future developments of BeiDou global navigation satellite system. Satell. Navig..

[11] ISO 17572-1:2015—Intelligent Transport Systems (ITS)—Location Referencing for Geographic Databases—Part 1: General Requirements and Conceptual Model. https://www.iso.org/standard/63400.html.

[12] ISO 17572-2:2018—Intelligent Transport Systems (ITS)—Location Referencing for Geographic Databases—Part 2: Pre-Coded Location References (Pre-coded Profile). https://www.iso.org/standard/69468.html.

[13] Groves P.D. (2011). Shadow matching: A new GNSS positioning technique for urban canyons. J. Navig..

[14] Kok M., Hol J.D., Schön T.B. (2017). Using inertial sensors for position and orientation estimation. arXiv.

[15] Lee N., Ahn S., Han D. (2018). AMID: Accurate magnetic indoor localization using deep learning. Sensors.

[16] Jiménez A., Seco F. (2005). Ultrasonic Localization Methods for Accurate Positioning.

[17] Fu Q., Yu H., Wang X., Yang Z., Zhang H., Mian A. (2020). FastORB-SLAM: A fast ORB-SLAM method with Coarse-to-Fine descriptor independent keypoint matching. arXiv.

[18] Campos C., Elvira R., Rodríguez J.J.G., Montiel J.M., Tardós J.D. (2021). ORB-SLAM3: An Accurate Open-Source Library for Visual, Visual–Inertial, and Multimap SLAM. IEEE Trans. Robot..

[19] Mur-Artal R., Montiel J.M.M., Tardos J.D. (2015). ORB-SLAM: A versatile and accurate monocular SLAM system. IEEE Trans. Robot..

[20] Davison A.J., Reid I.D., Molton N.D., Stasse O. (2007). MonoSLAM: Real-time single camera SLAM. IEEE Trans. Pattern Anal. Mach. Intell..

[21] Hess W., Kohler D., Rapp H., Andor D. Real-time loop closure in 2D LIDAR SLAM. Proceedings of the 2016 IEEE International Conference on Robotics and Automation (ICRA).

[22] Zhang J., Singh S. LOAM: Lidar Odometry and Mapping in Real-time. Proceedings of the Robotics: Science and Systems.

[23] Deschaud J.E. IMLS-SLAM: Scan-to-model matching based on 3D data. Proceedings of the 2018 IEEE International Conference on Robotics and Automation (ICRA).

[24] Ye H., Chen Y., Liu M. Tightly coupled 3d lidar inertial odometry and mapping. Proceedings of the 2019 International Conference on Robotics and Automation (ICRA).

[25] Leitinger E., Meyer F., Tufvesson F., Witrisal K. Factor graph based simultaneous localization and mapping using multipath channel information. Proceedings of the 2017 IEEE International Conference on Communications Workshops (ICC Workshops).

[26] Dellaert F. (2012). Factor Graphs and GTSAM: A Hands-On Introduction.

[27] Whelan T., Kaess M., Fallon M., Johannsson H., Leonard J., McDonald J. Kintinuous: Spatially Extended Kinectfusion. Proceedings of the RSS’12 Workshop on RGB-D: Advanced Reasoning with Depth Cameras.

[28] Wolcott R.W., Eustice R.M. Fast LIDAR localization using multiresolution Gaussian mixture maps. Proceedings of the 2015 IEEE International Conference on Robotics and Automation (ICRA).

[29] Wang L., Zhang Y., Wang J. (2017). Map-based localization method for autonomous vehicles using 3D-LIDAR. IFAC-PapersOnLine.

[30] Yoneda K., Tehrani H., Ogawa T., Hukuyama N., Mita S. Lidar scan feature for localization with highly precise 3-D map. Proceedings of the 2014 IEEE Intelligent Vehicles Symposium Proceedings.

[31] Cao V.H., Chu K., Le-Khac N.A., Kechadi M.T., Laefer D., Truong-Hong L. Toward a new approach for massive LiDAR data processing. Proceedings of the 2015 2nd IEEE International Conference on Spatial Data Mining and Geographical Knowledge Services (ICSDM).

[32] Biber P., Straßer W. The normal distributions transform: A new approach to laser scan matching. Proceedings of the 2003 IEEE/RSJ International Conference on Intelligent Robots and Systems (IROS 2003) (Cat. No. 03CH37453).

[33] Javanmardi E., Javanmardi M., Gu Y., Kamijo S. Autonomous vehicle self-localization based on multilayer 2D vector map and multi-channel LiDAR. Proceedings of the 2017 IEEE Intelligent Vehicles Symposium (IV).

[34] Shan T., Englot B., Meyers D., Wang W., Ratti C., Rus D. Lio-sam: Tightly-coupled lidar inertial odometry via smoothing and mapping. Proceedings of the 2020 IEEE/RSJ International Conference on Intelligent Robots and Systems (IROS).

[35] Im J.H., Im S.H., Jee G.I. (2016). Vertical corner feature based precise vehicle localization using 3D LIDAR in urban area. Sensors.

[36] Zheng H., Wang R., Xu S. (2016). Recognizing street lighting poles from mobile LiDAR data. IEEE Trans. Geosci. Remote Sens..

[37] High Definition Road Map of Seoul. http://map.ngii.go.kr/mn/mainPage.do.

[38] Nickolls J., Dally W.J. (2010). The GPU computing era. IEEE Micro.

[39] Harris M. (2007). Optimizing parallel reduction in CUDA. Nvidia Dev. Technol..

[40] Sanders J., Kandrot E. (2010). CUDA by Example: An Introduction to General-Purpose GPU Programming.

[41] Magnusson M. (2009). The Three-Dimensional Normal-Distributions Transform: An Efficient Representation for Registration, Surface Analysis, and Loop Detection. Ph.D. Thesis.

[42] Rusu R.B., Cousins S. 3D is here: Point Cloud Library (PCL). Proceedings of the IEEE International Conference on Robotics and Automation (ICRA).

[43] pcl::IterativeClosestPoint Class Template Reference. https://pointclouds.org/documentation/classpcl_1_1_iterative_closest_point.html.

[44] pcl::NormalDistributionsTransform Class Template Reference. https://pointclouds.org/documentation/classpcl_1_1_normal_distributions_transform.html.

[45] Caesar H., Bankiti V., Lang A.H., Vora S., Liong V.E., Xu Q., Krishnan A., Pan Y., Baldan G., Beijbom O. (2019). nuScenes: A multimodal dataset for autonomous driving. arXiv.

